# Real-Time Detection of Tsunami Ionospheric Disturbances with a Stand-Alone GNSS Receiver: A Preliminary Feasibility Demonstration

**DOI:** 10.1038/srep46607

**Published:** 2017-04-21

**Authors:** Giorgio Savastano, Attila Komjathy, Olga Verkhoglyadova, Augusto Mazzoni, Mattia Crespi, Yong Wei, Anthony J. Mannucci

**Affiliations:** 1 Department of Civil, Building and Environmental Engineering, University of Rome, La Sapienza, Rome, Italy; 2 Ionospheric and Atmospheric Remote Sensing Group, Jet Propulsion Laboratory, California Institute of Technology, Pasadena, California, USA; 3 Pacific Marine Environmental Laboratory, National Oceanic & Atmospheric Administration (NOAA), Seattle, WA, USA; 4 Joint Insititute for the Study of Atmosphere and Ocean (JISAO), University of Washington, Seattle, WA, USA

## Abstract

It is well known that tsunamis can produce gravity waves that propagate up to the ionosphere generating disturbed electron densities in the E and F regions. These ionospheric disturbances can be studied in detail using ionospheric total electron content (TEC) measurements collected by continuously operating ground-based receivers from the Global Navigation Satellite Systems (GNSS). Here, we present results using a new approach, named VARION (Variometric Approach for Real-Time Ionosphere Observation), and estimate slant TEC (sTEC) variations in a real-time scenario. Using the VARION algorithm we compute TEC variations at 56 GPS receivers in Hawaii as induced by the 2012 Haida Gwaii tsunami event. We observe TEC perturbations with amplitudes of up to 0.25 TEC units and traveling ionospheric perturbations (TIDs) moving away from the earthquake epicenter at an approximate speed of 316 m/s. We perform a wavelet analysis to analyze localized variations of power in the TEC time series and we find perturbation periods consistent with a tsunami typical deep ocean period. Finally, we present comparisons with the real-time tsunami MOST (Method of Splitting Tsunami) model produced by the NOAA Center for Tsunami Research and we observe variations in TEC that correlate in time and space with the tsunami waves.

The notion that gravity waves generated by tsunami waves (even with wave heights of a few centimeters in deep ocean) can propagate upward in the atmosphere and ultimately cause perturbations in the total electron content (TEC) of the ionosphere was first established by Daniels^1^, and was theoretically further developed by Hines^2^,^3^. Peltier and Hines^4^ subsequently showed that these TEC variations can be detected through ionosonde measurements.

Using this solid foundation and the abundance of GPS observations, researchers have set out goals to develop models and establish observational systems to provide reliable tsunami forecasts before the actual tsunami waves reach coastlines. It has been demonstrated that effects of an ocean tsunami can potentially be remotely observed as traveling ionospheric disturbances (TIDs) produced by the gravity waves. These TIDs were detected using different methods of observation, including ground-GPS^5^,^6^,^7^, Jason-1 radar altimeter^8^,^9^, incoherent scatter radar (ISR) at Arecibo^10^ and space-based measurements^11^,^12^.

Additional investigations highlighted that the detection of tsunami-driven TIDs is not always straightforward since there exists several other causes for TIDs, such as intense or large-scale tropospheric weather^13^,^14^,^15^, geomagnetic and auroral activity^16^,^17^, earthquakes^18^,^19^,^20^, and even unknown mechanisms^21^. Therefore, the relationship between detected TIDs and those that are induced by a tsunami has to be proven, for example by verifying that the horizontal speed, direction and spectral bandwidth of the TIDs match that of the ocean tsunami^7^.

While there has been progress in experimental work, along with theoretical modeling of the interactions between the ocean surface, atmosphere, and ionosphere^8^,^22^,^23^,^24^,^25^, recent reviews presented by^26^,^27^ concluded that the core scientific problems regarding the nature of the coupling between the ocean and ionosphere are still not sufficiently understood. To provide actionable geophysical data on the inferred amplitude, period, and velocity of a tsunami, based on the estimated gravity wave-induced TIDs, a number of real-time TEC monitoring systems are being developed utilizing the present capabilities of GNSS technology and infrastructures. As a matter of fact, up to now TEC variations are routinely estimated and geolocated in a post-processing mode.

The existing tsunami warning systems currently rely on numerical modeling (as MOST) and buoy observations^7^. Since the 2003 Rat Islands tsunami, the NOAA Center for Tsunami Research (NCTR) has developed a real-time model-forecast methodology ingesting deep-ocean tsunami measurements into the MOST model to produce timely and accurate tsunami forecasts for potentially vulnerable U.S. coastal communities^28^,^29^,^30^,^31^,^32^. As a major model component of this forecast system, MOST has been used to develop a database of tsunami propagation model results for nearly 2,000 tsunami sources covering all known subducting zones on earth. Based on an inversion algorithm^33^, the forecast method rapidly estimates the tsunami source to obtain a best fit between the pre-computed tsunami propagation database and the real-time tsunami measurements supported by a global tsunameter system composed of 65 deep-ocean bottom pressure sensors. Ground-GNSS observations processed in real-time may have the potential to enhance the current system by independently providing the tsunami speed and amplitude.

We are presenting a new GNSS processing algorithm, named VARION (Variometric Approach for Real-Time Ionosphere Observation) that focuses on the real-time detection of the TIDs caused by tsunami atmospheric gravity waves (see Appendix A for the methodology). VARION is an open source, entirely Python-based software (https://github.com/giorgiosavastano/VARION). It was derived from the VADASE (Variometric Approach for Displacements Analysis Standalone Engine) algorithm that was successfully applied to estimate in a real-time scenario the ground velocities and displacements induced by several earthquakes (e.g. the Tohoku-Oki earthquake, USGS Mw 9.0, 11 March 2011, 05:46:24 UTC; the Emilia earthquake, USGS Mw 6.0, 20 May 2012, 02:03:52), using a stand-alone GNSS receiver^34^,^35^,^36^. The VADASE algorithm was later modified and applied to geometry-free combinations of GNSS carrier-phase measurements for estimating TEC variations. Using the VARION algorithm each dual-frequency GNSS receiver is expected to provide time series of real-time TEC variations in a stand-alone operational mode.

For the validation of the algorithm we consider ionosphere effects of the tsunami generated by the Magnitude 7.8 - Haida Gwaii earthquake, that occurred at 03:04:08 Universal Time (UT) on 28 October 2012, 207 km SW of Prince Rupert, Canada (USGS, 2012). We investigate gravity waves observed in the ionosphere in the Island of Hawaii more than 4000 km from the epicenter and reached by the tsunami about 5 hours later. The NCTR’s real-time estimate of the tsunami source was based on an inversion process to fit the pre-computed model database with the tsunami records from the closest tsunameter 46419. The tsunami source was estimated to have a rupture area of 200 × 50 km^2^ with an average slip of 3.6 m, giving an earthquake moment magnitude of 8.0. Based on this source estimation, the model results were rapidly assembled to provide a basin-wide model forecast of tsunami propagation, including the waves propagating through the Hawaiian Islands used for comparison with the sTEC perturbations in the present study.

The Datasets section introduces the data and methodology used in the investigation and the results are presented and discussed in the section Results and Discussion. In the Conclusion section we present our conclusions and additional details about the VARION approach are offered in Appendix A.

## Datasets

### GPS dataset

**Figure 1:**
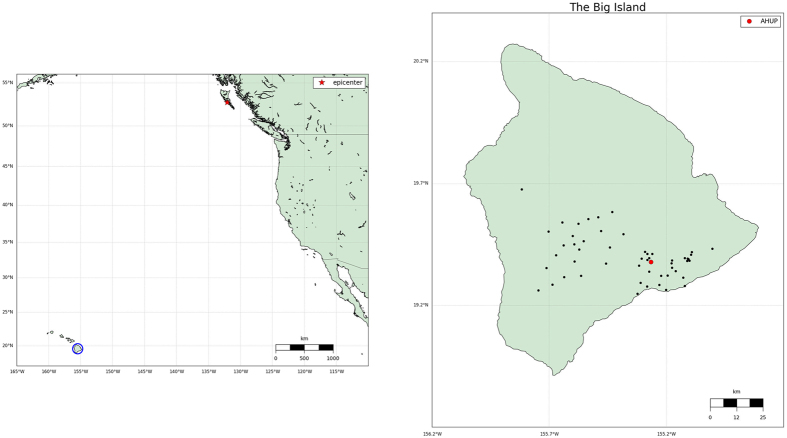
Map indicating the epicenter of the 10/27/2012 Haida Gwaii earthquake (left panel) and zoomed-in image of the Hawai’i Big Island, where all the 56 used GPS stations are located. The map has been generated using the matplotlib Basemap toolkit^55^.

To estimate the TEC variations we used the GPS observations collected at 56 Plate Boundary Observatory (PBO) sites located on the Hawaiian Islands (https://www.unavco.org/instrumentation/networks/status/pbo). All the GPS permanent stations are located on the Big Island (Hawai’i), (see Fig. 1). Observations were acquired at 15- and 30-seconds cadence.

### GPS processing

To process GPS observations we used two independent software packages. The VARION package based on single time differences of geometry-free combinations of GPS carrier-phase measurements, using a standalone GPS receiver and standard GPS broadcast products (orbits and clock corrections) that are available in real-time; and the bias-fixing Jet Propulsion Laboratory (JPL) technique that returns the absolute TEC values^37^, using a network approach to estimate the satellite and receiver interfrequency biases using the Global Ionospheric Mapping (GIM) software^38^.

### Tsunami model

For verifying the agreement of the estimated TEC variations with the tsunami, we used the real-time MOST (Method of Splitting Tsunami) model provided by the NOAA research center. The model employs a finite-difference approximation of the characteristic form of the shallow water wave equations by use of the splitting method^39^,^40^. For propagation, MOST uses the shallow water equations in spherical coordinates with numerical dispersion to account for different propagation wave speeds at different frequencies^41^. MOST uses the elastic model of Okada^42^ to compute the initial seafloor deformation resulting from a source model, which is then used directly as the initial deformation of the ocean surface in the model^43^.

## Results and Discussion

We processed the GPS observations from the 56 PBO GPS stations to estimate TEC variations at 15 s and 30 s rate with both VARION and JPL algorithms^37^. These two algorithms are designed to directly estimate two different parameters. As mentioned, the JPL algorithm is able to directly obtain the absolute TEC values, after estimating highly precise satellite and receiver Inter-Frequency Biases (IFBs) using about 200 GPS receivers distributed worldwide [e.g., refs 38, 44]. On the other hand, the direct outputs of the VARION algorithm are the sTEC variations (Equation 3), subsequently integrated over a certain time period; the variometric approach overcomes the problem of estimating the phase initial ambiguity and the IFBs, thus being ideal for real-time applications.

In order to highlight the TIDs, the TEC time series for each satellite were filtered to remove the TEC low-frequency variations (such as diurnal variations and multiple hour trends due to changing satellites elevation angles); VARION results were filtered using an 8th order polynomial, while the JPL algorithm uses a band-pass filter (0.5 to 5 mHz)^45^.

**Figure 2:**
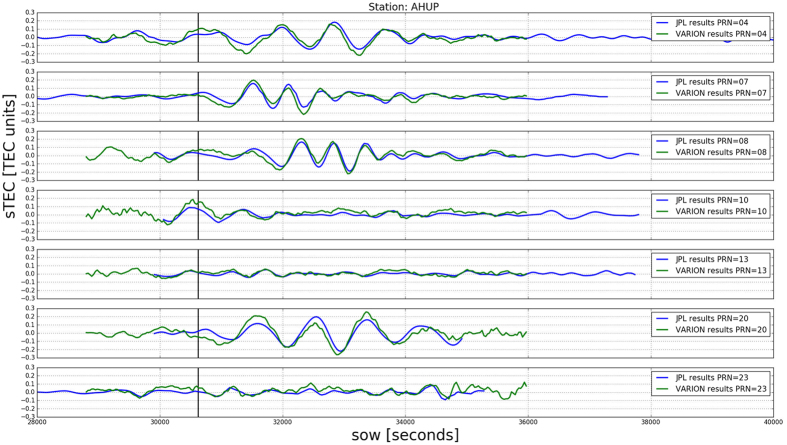
Comparison between TEC time series obtained from the VARION and JPL techniques. The TEC variations are computed for 7 satellites (PRNs 4, 7, 8, 10, 13, 20, 23) in view from the AHUP station on the Hawaiian Islands (latitude: 19.379 degrees, longitude: −155.266 degrees, height: 1104.881 meters). The black vertical line represents the time when the tsunami reached the Hawaiian Islands. TIDs were clearly detected, with good agreement between the two approaches.

Figure 2 shows the sTEC time series for two hours (08:00 to 10:00 UT - 28 October 2012) for 7 satellites in view from the AHUP station obtained with the two approaches. The vertical black line represents the time when the tsunami arrived at the Hawaiian Islands according to the MOST model. Very good agreement (RMS differences at the level of few hundredths TEC units) is evident, the differences are mostly due to the different data filtering methods applied. Moreover, we see significant TIDs for 5 satellites (PRNs 4, 7, 8, 10, 20) at different times due to the different locations of the ionospheric piercing points (IPPs) and the subionospheric points (SIPs : IPPs projections onto the ellipsoid). In particular, for the satellite PRN 10 the TEC perturbation occurred before the tsunami reached the Hawaiian Islands; this is in fact due to the geometry corresponding to the particular elevation and azimuth angles of satellite PRN 10 as the tsunami-generated TIDs were detected when tsunami the wave front was still about 150 km away from the coast. For satellites PRN 13 and 23 no significant TIDs were detected, likely due to elevations and azimutal positions with respect to the tsunami as a possible cause of the TIDs.

Can we detect TIDs induced by the tsunami itself ? If this could be confirmed, the potential of real-time detection of TIDs would be evident.

At first, we performed a wavelet analysis using the Paul wavelet (that gives better time localization than the Morlet one) and we determine both the dominant modes of variability and how those modes vary in time^46^. This technique allows us to highlight and evaluate the TID wave periods. Here, the wavelet analysis has been performed in python using scripts running the wavelet software provided by C. Torrence and G. Compo, and available at http://atoc.colorado.edu/research/wavelets/. We processed 260 sTEC time series, for all the satellites in view at the 56 GPS permanent stations. We found periods in the range of 10 to 30 minutes, similar to the periods of the tsunami ocean waves, which can range from 5 min up to an hour with the typical deep ocean period of only 10–30 wavelengths around 400 km, and the velocity approximately 200 m/s^4^.

**Figure 3:**
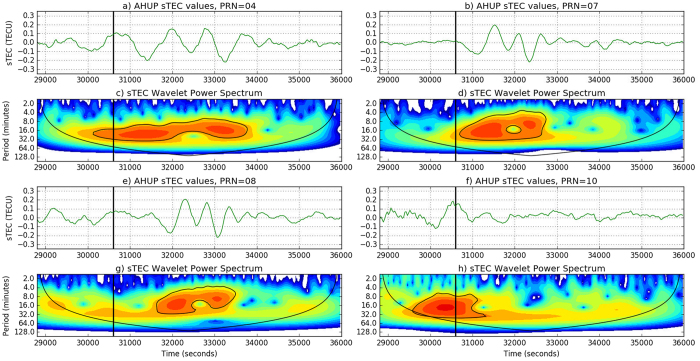
(**a,b,e,f**) Four of 260 time series used for the wavelet analysis, station AHUP, satellite PRN 4, 7, 8, 10. (**c,d,g,h**) The wavelet power spectrum used the Paul wavelet. The vertical axis displays the Fourier period (in min), the horizontal axis is time (s). The black vertical line represents the time when the tsunami reached the Hawaiian islands. The color panels represent the intensity of the power spectrum; the black contour encloses regions of greater than 95% confidence for a red-noise process with a lag-1 coefficient of 0.72^46^; the external black line indicates the “one of influence”, the limit outside of which edge effects may become significant. We clearly see the increase of the power spectrum for periods between 10 and 30 minutes during the TIDs.

**Figure 4:**
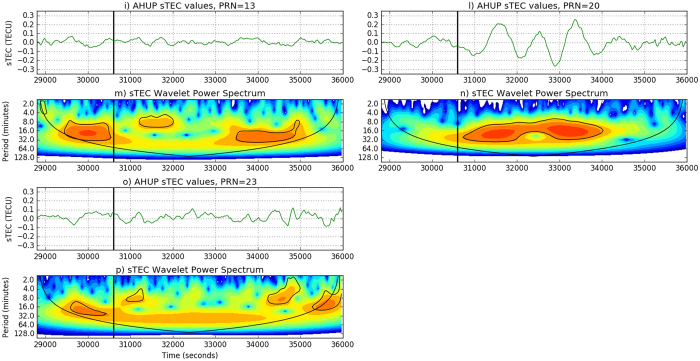
(**i,l,o**) Three of 260 time series used for the wavelet analysis, station AHUP, satellite PRN 13, 20, 23. (**m,n,p**) The wavelet power spectrum used the Paul wavelet. The vertical axis displays the Fourier period (in min), the horizontal axis is time (s). The black vertical line represents the time when the tsunami reached the Hawaiian islands. For satellites PRN 13 and 23 we do not see significant increase of the power spectrum for periods between 10 and 30 minutes during the TIDs.

Figures 3 and 4 show the sTEC time series wavelet analysis for the 7 satellites in view at the station AHUP. The upper panels show the sTEC time series obtained with the VARION software in a real-time scenario, as plotted in Fig. 2. The bottom panels indicate the wavelet spectra. The colors represent the intensity of the power spectrum and the black contour encloses regions of greater than 95% of confidence for a red noise process. We can identify 5 satellites (PRNs 4, 7, 8, 10, 20) with peaks consistent in time and period with the tsunami ocean waves. These results clearly show TIDs appearing after the tsunami reached the islands.

**Figure 5:**
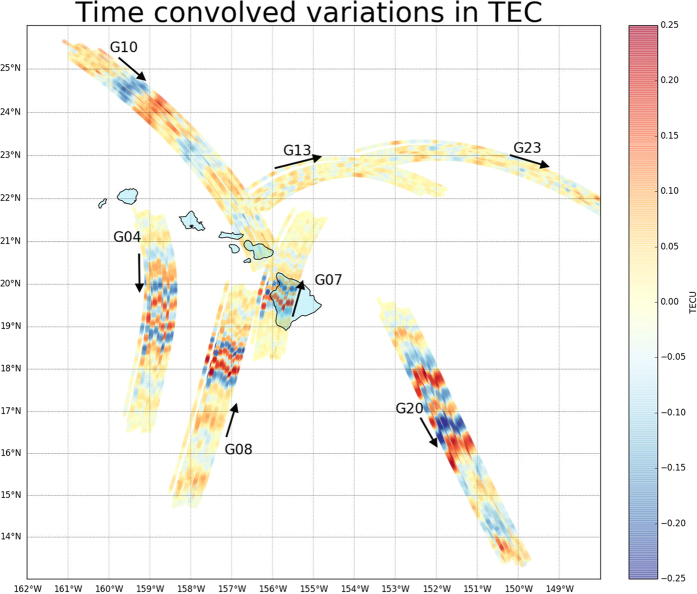
Space–time sTEC variations for two hours (08:00 to 10:00 UT – 28 October 2012 – cut–off angle set to 18°) at the SIPs (same positions of the corresponding IPPs on the map) for the 7 satellites seen from the 56 Hawaiian Hawaii Islands GPS permanent stations, after the Haida Gwaii earthquake. The TIDs are clearly visible in the interval of significant sTEC variations (from positive to negative values and vice-versa). It is also shown that PRN 10 detected TIDs prior to the tsunami arrival at Hawaiian Islands (08:30:08 UT). The map has been generated using the matplotlib Basemap toolkit^55^.

Figure 5 displays a map of the region around the Hawaiian Islands, the area of our focus in order to highlight the most significant sTEC variations. The colored tracks show the positions of the SIPs (equal to corresponding IPPs, when seen on the map) for each of the 7 satellites considered in Fig. 1 as seen from the 56 GPS permanent stations during an observation span of two hours (8:00 to 10:00 UT, 28 October 2012). The colors represent the variation in sTEC, obtained by VARION processing; the TIDs are clearly visible in the interval of significant sTEC variations (from positive to negative values and vice-versa).

**Figure 6:**
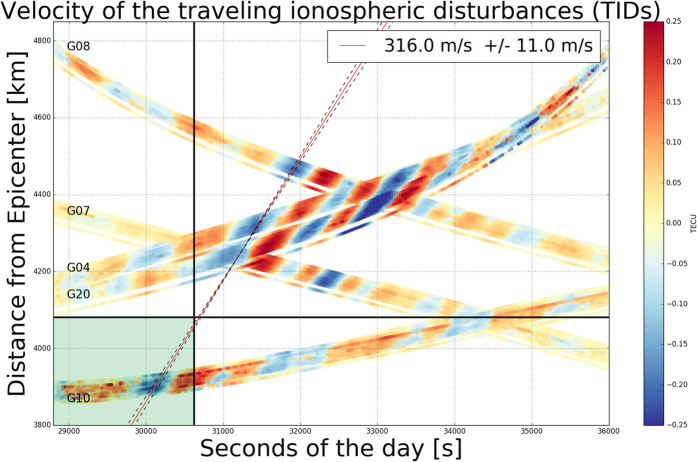
sTEC variations for two hours (08:00 to 10:00 UT – 28 October 2012) at the IPPs vs. distance from the Haida Gwaii earthquake epicenter, for the 7 satellites observed from the 56 Hawaii Hawaiian Islands GPS permanent stations. The TIDs are clearly visible in the interval of significant sTEC variations (from positive to negative values and vice-versa). The vertical and horizontal black lines represent the time (when the tsunami arrived at the Hawaiian Islands) and the distance (between the epicenter and the Big Island), respectively; it is evident that PRN 10 detected TIDs before the tsunami arrived at Hawaiian Hawaii Islands (08:30:08 UT). The slope of the straight line fitted, considering a linear least-squares regression for corresponding sTEC minima for different satellites, represent the TIDs mean propagation velocity.

Figure 6 shows time sTEC variations for two hours (08:00 to 10:00 UT - 28 October 2012) at the IPPs vs. distance from the Haida Gwaii earthquake epicenter, for the same 7 satellites under consideration. The TIDs are clearly visible in the interval of significant sTEC variations (from positive to negative values and vice-versa). The vertical and horizontal black lines represent the time (when the tsunami arrived at the Hawaiian Islands) and the distance (between the epicenter and the Big Island), respectively. In this way, we identify the green rectangle as the alert area and it is evident that satellite PRN 10, the closest to the earthquake epicenter detected TIDs before the tsunami arrived at Hawaiian Islands (08:30:08 UT). In the distance vs time plots (also called hodochrons) the slope of the straight line, fitted considering corresponding sTEC minima for different satellites, represents the horizontal speed estimate of TIDs. This plot indicates that the linear least-squares estimated speed of the TIDs is about 316 m/s and it is found to be in good agreement with a typical speed of the tsunami gravity waves estimated with ground-based GNSS receivers (see Appendix B). We note that such speed determinations via hodochron are not available in real-time, but neither are these estimates needed for real-time tsunami detection.

**Figure 7:**
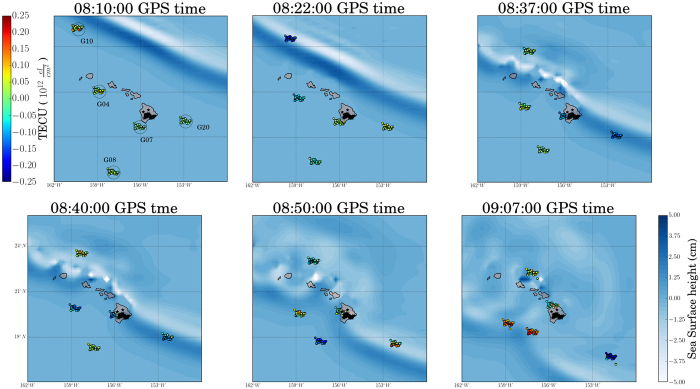
Space-time sTEC variations at 6 epochs within the two hours interval (08:00 to 10:00 UT – 28 October 2012) at the SIPs for the 5 satellites showing TIDs, overplotted the tsunami MOST model. TIDs are consistent in time and space with the tsunami waves. The maps have been generated using the matplotlib Basemap toolkit^55^.

Figure 7 displays a sequence of maps of the region around the Hawaiian Islands showing the variations in sTEC (determinable in real-time) at IPP/SPI locations on top of the MOST model sea-surface heights. Note that, just as the MOST model wavefronts are moving past the IPPs, the sTEC variations in the region become pronounced, correlated with the passage of the ocean tsunami itself. In particular, at 08:22:00 GPS time (08:21:44 UT) we are able to see sTEC perturbations from 56 stations looking at satellite PRN 10. The propagation of the MOST modeled tsunami passes the ionospheric pierce points located NW of the Big Island and offers insight with regard to the ionospheric response to the tsunami-driven atmospheric gravity wave. These perturbations are detected before the tsunami reached the islands as seen from the locations of the SIP points. The following frames indicate the tsunami-driven TIDs detected from the other 4 satellites (PRNs 4, 7, 8, 20) tracking the propagating tsunami (see supplementary video SV1).

## Conclusion

We have found observational evidence of variations in GPS sTEC measurements in the range of 0.1–0.2 TEC units (on the order of 1% of the background TEC value) that are associated with the Haida Gwaii tsunami of 28 October 2012. We compared two independent signal processing techniques, one available in real-time and one available in post-processing, and a good agreement was found between the JPL (post-processed) and VARION (real-time capable) results. We performed a wavelet analysis and we observed sTEC variations with a typical period between 10 and 30 minutes, consistent with the ocean tsunami waves. We estimated the speed of the TIDs generated by the tsunami-driven IGWs and we found a typical speed of about 316 km/s. From the comparison between the MOST model results and TECs measurements we have validated our results in time and space. Using signals from 56 GPS stations located on the Hawaiian Islands, we have detected TEC perturbations before the actual tsunami arrival; this is due to the geometry of the satellite PRN 10 (elevation and azimuth angles) and so the position of the SIPs at that time.

We have demonstrated that the real-time capable VARION algorithm is able to detect the TIDs generated by tsunami-driven gravity waves and may be considered as a novel contribution to future integrated operational tsunami early warning systems.

We are currently implementing the VARION algorithm in JPL’s Global Differential GPS system (GDGPS) providing real-time access to 1-Hz data streams from about 230 global real-time stations collecting data from multiple constellations including GPS, Galileo, GLONASS and BeiDou. With real-time streams of data the polynomial fit has been implemented as a finite duration impulse response (FIR) high-pass filter. For our application, a FIR filter is designed with 2048 coefficients (taps), and so when using 1-Hz data there is an initial delay of 35 minutes (2048/3600*60). This period is called the transitory phase (TP). When TP ends, the system will proceed in stationary phase (SP) and will provide continuous real-time estimates of TEC perturbations. This initial delay will not be expected to affect the reliability of the system because it occurs once the receiver starts tracking the particular satellite and this will likely not coincide with start of the event.

A real-time tsunami detection system could be designed using VARION combined with real-time data from different sources (e.g. seismometers, buoys, GNSS receivers). Once an earthquake is detected in a specific location, such a system will begin processing the real-time TEC outputs using multiple stations located near the epicenter searching for ionospheric signals that may be correlated with the expected tsunami propagation. The measurements would be collected and processed by a central processing facility also providing risk assessments and maps related to a particular earthquake event. The use of multiple independent data types will be expected to contribute significantly to the robustness of the system.

### Appendix A: VARION Methodology

The VARION approach is based on single time differences of geometry-free combinations of GPS carrier-phase measurements, using a standalone GPS receiver and standard GPS broadcast products (orbits and clocks corrections) that are available in real-time. We start from the carrier-phase observation, which in length units is


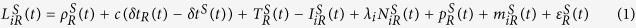


where *i* is the index of the signal frequency, subscript R refers to a particular receiver and superscript S refers to a satellite. *λ* is the carrier phase wavelength; 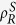 is the geometric range; *c* is the speed of light; *δt*_*R*_ and *δt*^*S*^ are the receiver and the satellite clock errors, respectively; 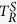 and 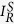 are the tropospheric and the ionospheric delays along the path from the satellite to the receiver, respectively; 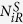 is the phase ambiguity; 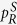 is the sum of the other effects (relativistic effects, phase center variations, and phase windup); and 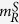 and 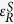 represent the multipath and the noise, respectively.

If no cycle slips occurred, the unknown carrier phase ambiguity can be considered constant between two consecutive epochs. The receiver and the satellite Inter-Frequency Biases (IFB) in the carrier-phase ionospheric obsevable are also assumed as constant for a given period^47^,^48^,^49^,^50^. For these reasons, differentiating (Equation 1) in time between two consecutive epochs (t and t + 1), and applying the geometry-free combination, we obtain the geometry-free time single-difference observation equation (Equation 2), with no need of estimate in real-time the phase ambiguity and the IFB.


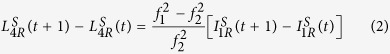


where the subscript 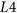 refers to the geometry-free combination and *f*_1_ and *f*_2_ are the *L*1 and *L*2 frequencies, 1575.42 MHz and 1227.60 MHz respectively.

Taking into account the ionospheric refraction along the geometric range, we compute the TEC variations between two consecutive epochs (Equation 3)


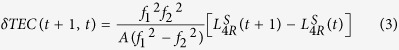


Subsequently, the TEC variations are integrated over the time interval (from 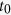 to 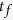) during which the tsunami event occurred, to retrieve tsunami ionospheric disturbances (Equation 4).


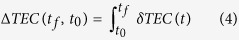


We normally consider time intervals of 2 hours. In order to remove longer period variations in TEC time series (such as diurnal variations and multiple-hour trends due to changing elevation angle of the receiver-satellite line-of-sight), we used an 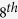 order polynomial to fit the TEC time series, and subtract the observed TEC values from the polynomial fit, with the residuals representing the variation in TEC due to a TID perturbation (see ref. 51 for detailed methodology).

Concerning the geolocation of the TEC estimates, the VARION algorithm uses the Klobuchar broadcast ionospheric model, based on an ionosphere thin shell (in our case, at the height of 350 km) approximation^52^, enabling the real-time computation of both the positions of the ionospheric pierce points and the sub-ionospheric points (SIP: IPP projection onto the ellipsoid).

It is useful to underline that we considered the already presented technique based on double time differences of VTEC with intervals of 300 seconds^53^,^54^ and we believe that the main advantages of our VARION algorithm are the independence from cycle-slips (they are detected and removed as outliers), the true real-time TEC estimations and the very low computational time.

### Appendix B: Ionospheric Height vs TID Velocity

**Figure 8:**
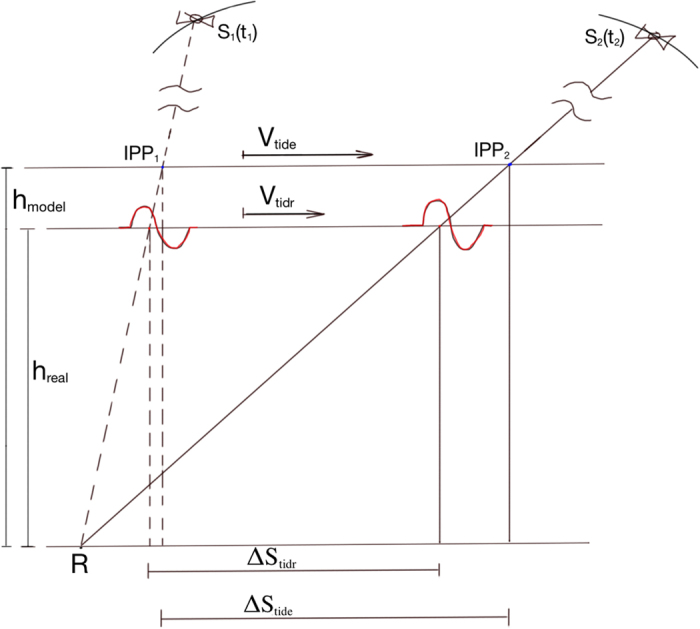
Schematic representation of the TID detection at *t*_1_ and *t*_2_ = *t*_1 _+ Δ*t* by two different satellites *S*_1_ and *S*_2_. *R* represents the receiver, *h*_*model*_ and *h*_*real*_ represent the modeled ionospheric layer and the real ionospheric layer. In this case the two layers are located at 300 and 350 km, respectively.

Under the flat Earth approximation, it is possible to show that there is a direct dependence of the TID estimated speed on the modeled ionospheric layer height. Taking into account Fig. 8, where the subscripts 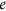 and 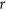 represent estimated and real values, the estimated TID speed 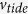 using ground-based GNSS observations will not necessary be equal to the real TID speed 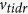. The two values are directly proportional as shown in Eqn. 5.


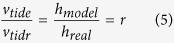


where 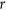 represents the ratio between the modeled ionospheric height layer the height at which we have the maximum of the electron density, respectively.

In particular, the ionospheric shell height used in this case has a constant value of 350 km while the electron density profiles obtained using, e.g., the International Reference Ionosphere (IRI), a standard empirical model of the global ionosphere (available at http://omniweb.gsfc.nasa.gov/vitmo/iri_vitmo.html), indicates a maximum electron density value to occur at 300 km (see Supplementary Figure SF1). If we consider the TID speed to be almost equal to the tsunami speed, we can conclude that the best TID speed estimation using GNSS observations will be around 300 m/s.

## Additional Information

**How to cite this article**: Savastano, G. *et al*. Real-Time Detection of Tsunami Ionospheric Disturbances with a Stand-Alone GNSS Receiver: A Preliminary Feasibility Demonstration. *Sci. Rep.* **7**, 46607; doi: 10.1038/srep46607 (2017).

**Publisher's note:** Springer Nature remains neutral with regard to jurisdictional claims in published maps and institutional affiliations.

## Supplementary Material

Supplementary Information

Supplementary Video S1
