# Supplementary material for: Real-Time Detection of Tsunami Ionospheric Disturbances with a Stand-Alone GNSS Receiver: A Preliminary Feasibility Demonstration
Source: Sci Rep. 2017 Apr 21;7:46607. doi: 10.1038/srep46607 (PMC5399489; doi:10.1038/srep46607)
Supplement: Supplementary Information [file srep46607-s1.pdf]

Real-Time Detection of Tsunami Ionospheric Disturbances with a Stand-Alone GNSS Receiver: A Preliminary Feasibility Demonstration

Giorgio Savastano[1,*], Attila Komjathy[2], Olga Verkhoglyadova[2], Augusto Mazzoni[1], Mattia Crespi[1], Yong Wei[3,4], and Anthony J. Mannucci[2]

[1]Department of Civil, Building and Environmental Engineering, University of Rome, La Sapienza, Rome, Italy.
[2]Ionospheric and Atmospheric Remote Sensing Group, Jet Propulsion Laboratory, California Institute of Technology, Pasadena, California, USA.
[3]Pacific Marine Environmental Laboratory, National Oceanic & Atmospheric Administration (NOAA), Seattle, WA, USA
[4]Joint Insititute for the Study of Atmosphere and Ocean (JISAO), University of Washington, Seattle, WA, USA
[*]giorgio.savastano@uniroma1.it

ABSTRACT

Supplementary Information

Supplementary Info

The supplementary video SV1 demonstrates that in our assessment, an excellent agreement is achieved both in space and time between the ionospheric measurements obtained with our VARION algorithm in a real-time scenario and the real-time tsunami model MOST (Method of Splitting Tsunami) provided by the NOAA Center for Tsunami Research. In the video, indeed 56 receivers are used to plot ionospheric TEC perturbations generated by the tsunami and the real-time MOST model. In particular, at 08:22:00 GPS time (08:21:44 UT) we are able to see sTEC perturbations from 56 stations looking at satellite PRN 10. The propagation of the MOST modeled tsunami passes the ionospheric pierce points located NW of the Big Island and offers insight with regard to the ionospheric response to the tsunami-driven atmospheric gravity wave. These perturbations are detected before the tsunami reached the islands as seen from the locations of the SIP points. The following frames indicate the tsunami-driven TIDs detected from the other 4 satellite (PRNs 4,7,8,20) tracking the propagating tsunami. This video has been generated converting a series of image frames into a video format file using the ImageMagick (v. 7.0.4-8) command-line tools (available at `http://imagemagick.org/script/index.php`). The map has been generated using the matplotlib Basemap toolkit (Hunter, 2007 [55]).

The supplementary figure SF1 shows the electron density profile obtained with the International Reference Ionosphere (IRI), a standard empirical model of the global ionosphere (available at `http://omniweb.gsfc.nasa.gov/vitmo/iri_vitmo.html`), indicates a maximum electron density value to occur at 300 km.

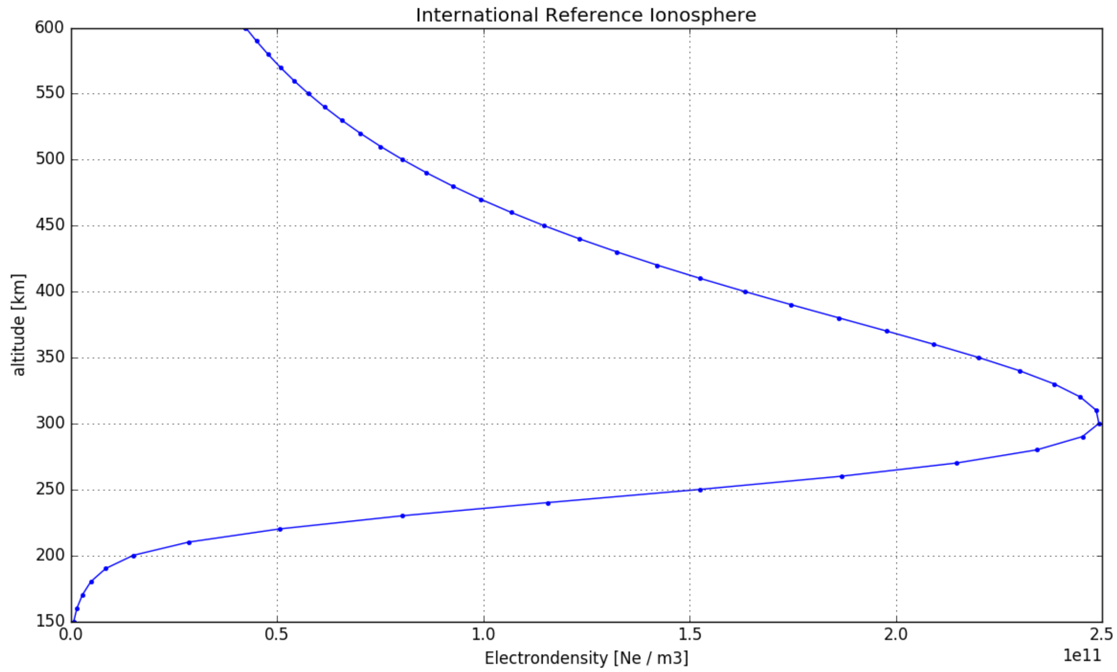

Figure 1. Electron density profile obtained with the IRI model computed at the Hawai'i Big Island (10:00 UT − 28 October 2012). The model indicates a maximum electron density value to occur at 300 km.
